# Antipsychotic prescribing patterns and determinants in first-episode psychosis: a 2019–2024 cross-sectional study from Zambia

**DOI:** 10.1186/s12888-026-07847-y

**Published:** 2026-01-30

**Authors:** Lihlizulu Thabo Moyo, Thandiwe Martha Tembo, Brian Maila

**Affiliations:** 1 Chainama Hills College Hospital, P.O. Box 30043, Lusaka, Zambia; 2 Division of Global Public Health, University of California, San Diego, 9500 Gilman Drive, La Jolla, CA 92093 USA; 3 School of Social Work, San Diego State University, 5500 Campanile Drive, San Diego, San Diego, CA 92182 USA

**Keywords:** Zambia, First-episode psychosis, Antipsychotics, Prescribing patterns, First-generation antipsychotics, Second-generation antipsychotics, Determinants

## Abstract

**Background:**

First-episode psychosis represents a critical period in psychotic disorders, during which timely, appropriate treatment can greatly influence long-term outcomes. Antipsychotics remain the foundation of first-episode psychosis management, yet prescribing patterns vary across settings due to patient characteristics, clinical factors, and resource availability. Although factors influencing antipsychotic choice are increasingly recognized, these have not been examined in the Zambian context. Understanding local prescribing practices is essential to optimizing care and outcomes for individuals with first-episode psychosis.

**Objective:**

To describe antipsychotic prescription patterns in adults with first-episode psychosis and identify demographic and treatment-related factors associated with second-generation antipsychotic use.

**Methods:**

A cross-sectional review of medical records was conducted among 213 adults (≥ 18 years) prescribed an antipsychotic for First-episode psychosis between 2019 and 2024 at Chainama Hills College Hospital in Lusaka, Zambia. A data abstraction form captured sociodemographic, clinical, and prescriber information. Descriptive statistics characterized the sample and prescribing patterns, and multivariable logistic regression identified factors independently associated with prescribing second-generation versus first-generation antipsychotics.

**Results:**

The mean age of participants was 30.23 years (SD = 11.47), with 72.3% being male, 65.3% single, and 62.0% unemployed. Other psychoses (54.0%) and brief/acute psychosis (34.7%) were the most common diagnoses in our sample. Chlorpromazine (*n* = 74) and haloperidol (*n* = 75), both first-generation agents, were the most frequently prescribed, followed by risperidone (*n* = 65), a second-generation agent. Overall, first-generation antipsychotics accounted for a slightly larger share of prescriptions. In multivariable logistic regression analysis, females had higher odds of receiving a second-generation antipsychotic prescription (AOR = 7.55, 95% CI 3.27–17.41, *p* < 0.001), while partial adherence to treatment reduced the odds of second-generation antipsychotic prescription (AOR = 0.39, 95% CI 0.16–0.92, *p* < 0.05).

**Conclusion:**

The findings indicate a relative preference for first-generation antipsychotics, with prescribing influenced by gender and treatment adherence. Prescribing reflects pragmatic, context-driven decision-making shaped by medication availability and affordability in a low-resource setting. This calls for policies that strengthen evidence-based prescribing practices, integrate sex-sensitive and adherence-focused approaches, and improve access to effective antipsychotic options within health systems.

**Clinical trial number:**

Not applicable. This study is a secondary analysis of de-identified electronic health records and does not constitute a clinical trial.

**Supplementary Information:**

The online version contains supplementary material available at 10.1186/s12888-026-07847-y.

## Background

First episode psychosis (FEP) refers to the first occurrence of psychotic symptoms in an individual, typically during adolescence or early adulthood [1]. Psychosis is a mental health condition characterized by a disconnection from reality, and is characterized by symptoms such as hallucinations, delusions, disorganized thinking, bizarre behaviour and impaired insight [2]. First-episode psychosis (FEP) represents a critical window in the course of psychotic disorders, during which early and appropriate intervention can substantially influence long-term clinical, functional, and social outcomes [3]. Decisions made at the onset of treatment—including the choice of antipsychotic medication—have enduring implications for symptom remission, treatment adherence, side-effect burden, and sustained engagement with mental health services [4]. Consequently, optimizing pharmacological management during FEP has become a central focus of international clinical guidelines and early-intervention models.

Antipsychotic medications are commonly categorized into first-generation antipsychotics (FGAs) and second-generation antipsychotics (SGAs) based on their pharmacological profiles. FGAs (e.g., haloperidol, chlorpromazine) primarily act through dopamine D2 receptor antagonism and are associated with a higher risk of extrapyramidal symptoms and tardive dyskinesia. SGAs (e.g., risperidone, olanzapine) combine dopamine D2 and serotonin 5-HT2A receptor antagonism, resulting in lower extrapyramidal risk but greater metabolic adverse effects [5–7]. Over the past two decades, many international guidelines, including those from the American Psychiatric Association and the National Institute for Health and Care Excellence, and recently the treatment guidelines for mental disorders in Zambia, have recommended SGAs as first-line agents for FEP, primarily due to their lower risk of extrapyramidal symptoms and tardive dyskinesia compared with FGAs [5, 8, 9]. However, the evidence base underlying these recommendations is nuanced. Meta-analyses and comparative effectiveness studies indicate that SGAs do not consistently demonstrate superiority over FGAs in terms of symptom reduction, relapse prevention, or long-term functional outcomes [7, 10]. Moreover, both antipsychotic classes are associated with substantial adverse-effect burdens and high rates of early treatment discontinuation in FEP, underscoring the complexity of medication selection during FEP care [10]. The existing studies suggest that guideline recommendations reflect trade-offs rather than unequivocal superiority of one drug class over another.

Beyond pharmacological considerations, antipsychotic prescribing in FEP is shaped by a multilevel interplay of patient-, clinician-, and system-level factors. Patient characteristics such as age, sex, symptom severity, comorbidities, and treatment adherence influence prescribing decisions due to differences in pharmacokinetics, tolerability, and clinical response [11–13]. Clinician-level factors, including training background, years of experience, and familiarity with specific agents, also contribute to variability in prescribing practices and guideline adherence [14]. At the health system level, medication availability, affordability, inclusion on national essential medicines lists, procurement mechanisms, and capacity for adverse-effect monitoring strongly influence treatment options, particularly in low- and middle-income countries (LMICs) [4, 15–17]. In such settings, prescribing patterns may diverge from international guideline recommendations not due to lack of clinical knowledge, but as pragmatic responses to structural and systemic constraints. From a global mental health and implementation science perspective, understanding how evidence-based recommendations are adapted within constrained health systems is essential for interpreting real-world treatment patterns and informing contextually appropriate policy and practice interventions. Framing prescribing practices as outcomes shaped by health system realities—rather than as deviations from guidelines—provides a more accurate and equitable lens through which to interpret care delivery in LMIC settings.

Chainama Hills College Hospital, located in Lusaka, Zambia, is the country’s largest tertiary psychiatric referral hospital and receives patients referred from across the nation. Mental health services in Zambia are delivered within a resource-constrained health system, where psychotropic medication availability and affordability vary, and national treatment guidelines—launched in 2022—generally recommend SGAs for psychotic disorders but do not provide FEP-specific algorithms [8]. In practice, both FGAs and SGAs are used in the management of FEP, with prescribing influenced by patient demographic characteristics, drug availability, cost, patient adherence, and clinician judgment.

Despite the growing global literature on FEP, evidence from Sub-Saharan Africa remains limited, particularly regarding real-world antipsychotic prescribing patterns and their determinants. In Zambia, no prior study has examined antipsychotic use among individuals with FEP or explored the contextual factors shaping the choice between FGAs and SGAs in routine clinical practice. Addressing this gap is critical for contextualizing guideline implementation, identifying structural barriers to optimal care, and informing mental health system strengthening efforts.

Accordingly, this study aimed to (1) describe antipsychotic prescribing patterns among adults with FEP at a tertiary psychiatric hospital in Zambia, and (2) identify demographic, clinical, and treatment-related factors associated with the prescription of second-generation versus first-generation antipsychotics. We hypothesized that SGA prescribing would be more likely among female patients and among patients with better documented treatment adherence, reflecting sex-based tolerability considerations and pragmatic prescribing strategies within the Zambian health system. By situating prescribing decisions within both the clinical evidence base and the structural realities of a resource-limited health system, this study contributes contextually grounded evidence to the global mental health literature on FEP treatment.

## Methods

### Study design and setting

This study employed a cross-sectional design using retrospective review of prescription charts and medical records to examine patterns and determinants of antipsychotic prescribing at Chainama Hills College Hospital, the largest tertiary psychiatric referral hospital in Lusaka, Zambia. The hospital provides specialized inpatient and outpatient mental health and addiction services. The institution receives referrals from across the country, serving a nationally diverse patient population.

### Study population and identification of first-episode psychosis

**Fig. 1:**
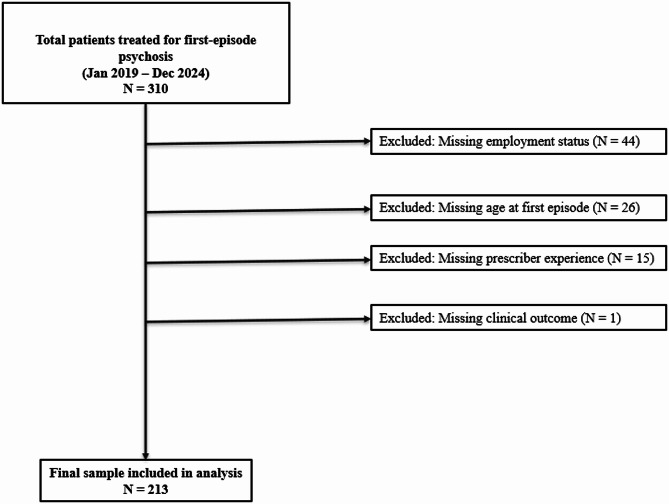
Participant Flow Diagram

The study included all adult patients (aged 18 years and older) who were prescribed at least one antipsychotic medication between January 2019 and December 2024 and had a documented diagnosis of first-episode psychosis in their medical records. FEP was identified based on documentation of a first-ever presentation of psychotic symptoms and initiation of antipsychotic treatment attributed to an ICD/DSM-5 psychiatric diagnosis, as recorded by the treating clinician in the medical chart. Age was defined at the time of the first antipsychotic prescription associated with the documented first psychotic episode. No patients under 18 years of age were identified in the dataset, likely reflecting historical referral of child and adolescent psychiatric cases to a separate tertiary facility prior to the recent expansion of services at Chainama Hills College Hospital. Patients were eligible for inclusion if they had a documented DSM-5 or ICD-10 psychiatric diagnosis and received treatment during the study period. Records with incomplete prescribing information or missing key demographic or clinical variables required for the analysis were excluded (e.g., adverse effects of the medications, presence of comorbidities) as this study followed a complete case analysis approach (Fig. 1).

### Data collection

Data was extracted from patient medical records using a standardized data collection form (Supplementary Table A). Variables collected included sociodemographic characteristics (age, sex, marital status, employment status, socioeconomic status), clinical characteristics (primary psychiatric diagnosis, symptom severity at presentation, type of antipsychotic prescribed [first-generation vs. second-generation], specific antipsychotic prescribed, and patient adherence level), prescriber characteristics (specialty and years of mental health experience), and patient adherence level. Data entry was performed in duplicate and cross-checked to ensure accuracy.

### Description of measures

#### Outcome of the study

The outcome variable was the type of antipsychotic prescribed, classified as first-generation antipsychotics (FGAs) (typical) and second-generation antipsychotics (SGAs) (atypical). This variable was managed as a binary variable, coded as FGAs [0] and SGAs [1]. When multiple prescriptions were recorded for a patient, only the first antipsychotic prescribed at the time of the initial documented psychotic episode was included in the analysis. There were no cases involving concurrent initiation of both FGAs and SGAs as polypharmacy at treatment initiation was uncommon in the medical/chart review. Hence, this variable was excluded from regression analyses.

### Independent variables

Independent variables included sociodemographic characteristics (age, sex, marital status, employment status, and socioeconomic status), clinical characteristics (primary psychiatric diagnosis, specific antipsychotic prescribed, and patient adherence level), and prescriber characteristics (specialty and years of experience in mental health practice). Age was analyzed as a continuous variable to describe the mean age of the sample and was additionally categorized into age groups for logistic regression analyses. Socioeconomic status was classified based on documented monthly household income in the medical records and categorized as low (< ZMW 2,000), middle (ZMW 2,000–9,200), and high (> ZMW 9,200). For both descriptive and multivariable logistic regression analyses, socioeconomic status was subsequently managed as a binary variable, defined as low (< ZMW 2,000) versus middle/high (≥ ZMW 2,000), to ensure adequate cell sizes and model stability. Patient adherence level was categorized based on clinician-documented assessments in the medical records. *Complete adherence* indicated consistent use of medication as prescribed, *partial adherence* indicated intermittent or inconsistent use, and *non-adherence* indicated documented discontinuation or refusal of medication. Medication adherence reflected clinician-documented assessments during the initial treatment period following first antipsychotic initiation, based on inpatient observation and/or early outpatient follow-up, rather than long-term adherence. Information on medical, substance use, and psychiatric comorbidities, as well as adverse effects, was inconsistently documented in the medical records, and information on hospitalizations related to treatment was not available. We did not plan to collect information on non-pharmacological interventions (e.g., psychotherapy), although such interventions are offered within the facility. Consequently, these variables were not included in the analyses.

### Statistical analysis

All analyses were conducted using **IBM SPSS Statistics (Version 30)**. Descriptive statistics were used to summarize the characteristics of the study population. Categorical variables were summarized using frequencies and percentages, while continuous variables were summarized using means and standard deviations. Bivariate analyses were performed to examine associations between independent variables and the type of antipsychotic prescribed (first-generation vs. second-generation). Pearson’s chi-square tests were used for categorical variables, and independent-samples *t*-s were used for continuous variables, as appropriate. Logistic regression analyses were conducted to identify factors associated with the prescription of second-generation antipsychotics. First, unadjusted (bivariate) logistic regression models were fitted for each independent variable. Variables were then entered into a multivariable logistic regression model to estimate adjusted odds ratios (AORs) and corresponding 95% confidence intervals (CIs). Independent variables were selected a priori based on prior literature demonstrating their relevance to antipsychotic prescribing decisions in FEP. These included demographic factors (age and sex), social characteristics (marital status and employment status), clinical presentation (primary psychiatric diagnosis and medication adherence), and prescriber-level characteristics (specialty and years of experience) [7, 10, 12, 14, 18]. Demographic factors such as age and sex influence pharmacokinetics, tolerability, and risk of adverse effects, which in turn shape prescribing choices [12, 13, 18]. Social characteristics, including marital and employment status, serve as proxies for social functioning and support, which are associated with treatment engagement and continuity of care [10, 18]. Clinical presentation, particularly primary diagnosis and medication adherence, is a key determinant of antipsychotic selection due to its association with relapse risk, treatment response, and need for adherence-enhancing strategies [10, 19]. Prescriber-level characteristics such as specialty and years of experience influence guideline adherence and clinical decision-making, contributing to variability in antipsychotic prescribing practices [12, 14]. Because information on comorbidities, adverse effects, hospitalizations related to treatment, and non-pharmacological interventions was incomplete or unavailable, these variables were not included in the models. Analyses were conducted using a complete-case approach. Statistical significance was defined as a two-sided *p* value of < 0.05.

### Ethical considerations

This study was executed as the initial segment of the parent investigation, “***Exploring Antipsychotic Prescription Patterns for First-Episode Psychosis: A Mixed-Methods Study at Chainama Hills College Hospital***,*** Lusaka***,*** Zambia***,” after obtaining ethical clearance from the University of Zambia Biomedical Research Ethics Committee (**UNZABREC Approval Number: REF. No. 5903 − 2024**), authorization from the National Health Research Authority (NHRA), and institutional consent from the management of Chainama Hills College Hospital to proceed with the study. In this retrospective investigation utilizing anonymized records, informed consent was waived for the patients whose records were reviewed. Confidentiality was preserved by de-identifying patient records during data extraction and analysis. The study was conducted in accordance with the ethical principles outlined in the Declaration of Helsinki. 

## Results

### Sociodemographic and clinical characteristics of the sample

Table 1 presents the demographic and clinical characteristics of the study population, stratified by type of antipsychotic prescribed (first-generation antipsychotics [FGA] vs. second-generation antipsychotics [SGA]). A total of 213 adult patients with first-episode psychosis met the inclusion criteria and were included in the analysis. The mean age of participants was 30.23 years (SD = 11.47), with the largest proportion falling in the 18–24 age group. Males comprised 72.3% of the sample. Over half of the sample reported being single (65.3%), Unemployed (62.0%), and middle/high socioeconomic status (77.9%). Other Types of Psychosis (Substance/Mood/Medical/PTSD/Other) were the most common primary diagnosis (54.0%), followed by Brief/Acute & Transient Psychosis (34.7%) and Schizophrenia spectrum Psychosis (11.3%). Majority of the patients received a prescription from a Clinical Officer – Psychiatry (COP)/General (COG) (91.1%), with over half of the patients receiving a prescription from prescribing practitioners with 2 to 5 years of experience working in the field of psychiatry and mental health (54,5%), and over half of the sample reporting having Complete Adherence to the medications prescribed (62.0). Patients differed significantly in characteristics, such as the mean age, the age group, sex, marital status, employment status, prescriber years of experience in mental health, and patient level of adherence by antipsychotic group prescribed (*p* < 0.05) on bivariate analysis. However, the sample did not differ significantly in terms of Socioeconomic Status, Primary Diagnosis, and Prescribing Practitioner Specialty by Antipsychotic group prescribed (*p* > 0.05).

**Table 1:** Characteristics of the sample (*N* = 213) by antipsychotic group (FGA vs. SGA)

Characteristic	Total *n* (%) or Mean (SD)	FGA *n* (%)	SGA *n* (%)	*p* value
**All**	213 (100)	150 (70.4)	63 (29.6)	-
**Age (Years**,** mean ± SD)**	30.23(± 11.47)	28.28(± 10.50)	34.87(± 12.405)	< 0.001
**Age Group (Years)**				
18–24	89 (41.8)	74 (49.3)	15 (23.8)	< 0.001
25–34	62 (29.1)	44 (29.3)	18(28.6)	
35–44	37 (17.4)	19 (12.7)	18 (28.6)	
45+	25 (11.7)	13(8.7)	12(19.0)	
**Sex**				
Male	154 (72.3)	128 (85.3)	26 (41.3)	< 0.001
Female	59 (27.7)	22 (14.7)	37 (58.7)	
**Marital Status**				
Single	139 (65.3)	111(74.0)	28(44.4)	< 0.001
Married	51 (23.9)	28(18.7)	23(36.5)	
Divorced/Widowed	23 (10.8)	11(7.3)	12(19.0)	
**Employment Status**				
Pupil/Student	27 (12.7)	25(16.7)	2(3.2)	< 0.001
Unemployed	132 (62.0)	93(62.0)	39(61.9)	
Self-Employed/Employed	54 (25.4)	32(21.3)	22(34.9)	
**Socioeconomic Status**				
Low (< ZMW 2,000)	47 (22.1)	30(20.0)	17(27.0)	> 0.05
Middle/High (≥ ZMW 2,000)	166 (77.9)	120(80.0)	46(73.0)	
**Primary Diagnosis**				
Brief/Acute & Transient Psychosis	74 (34.7)	47(31.3)	27(42.9)	> 0.05
Schizophrenia spectrum Psychosis	24 (11.3)	18(12.0)	6 (9.5)	
Other Types of Psychosis (Substance/Mood/Medical/PTSD/Other)	115 (54.0)	85(56.7)	30(47.6)	
**Prescribing Practitioner Specialty**				
Registrar – Psychiatry	19 (8.9)	14(9.3)	5(7.9)	> 0.05
Clinical Officer – Psychiatry (COP)/General (COG)	194 (91.1)	136(90.7)	58(92.1)	
**Prescriber Years of Experience in Mental Health**				
< 2 Years	14 (6.6)	10(6.7)	4(6.3)	< 0.05
2 to 5 Years	116 (54.5)	81(54.0)	35(55.6)	
> 5 Years	83 (39.0)	59(39.3)	24(38.1)	
**Patient Adherence Level**				
Complete Adherence	132 (62.0)	84(56.0)	48(76.2)	< 0.05
Non-adherence	18 (8.5)	15(10.0)	3(4.8)	
Partial Adherence	63 (29.6)	51(34.0)	12(19.0)	

### Antipsychotic prescribing patterns at a Zambian tertiary psychiatric hospital

**Fig. 2:**
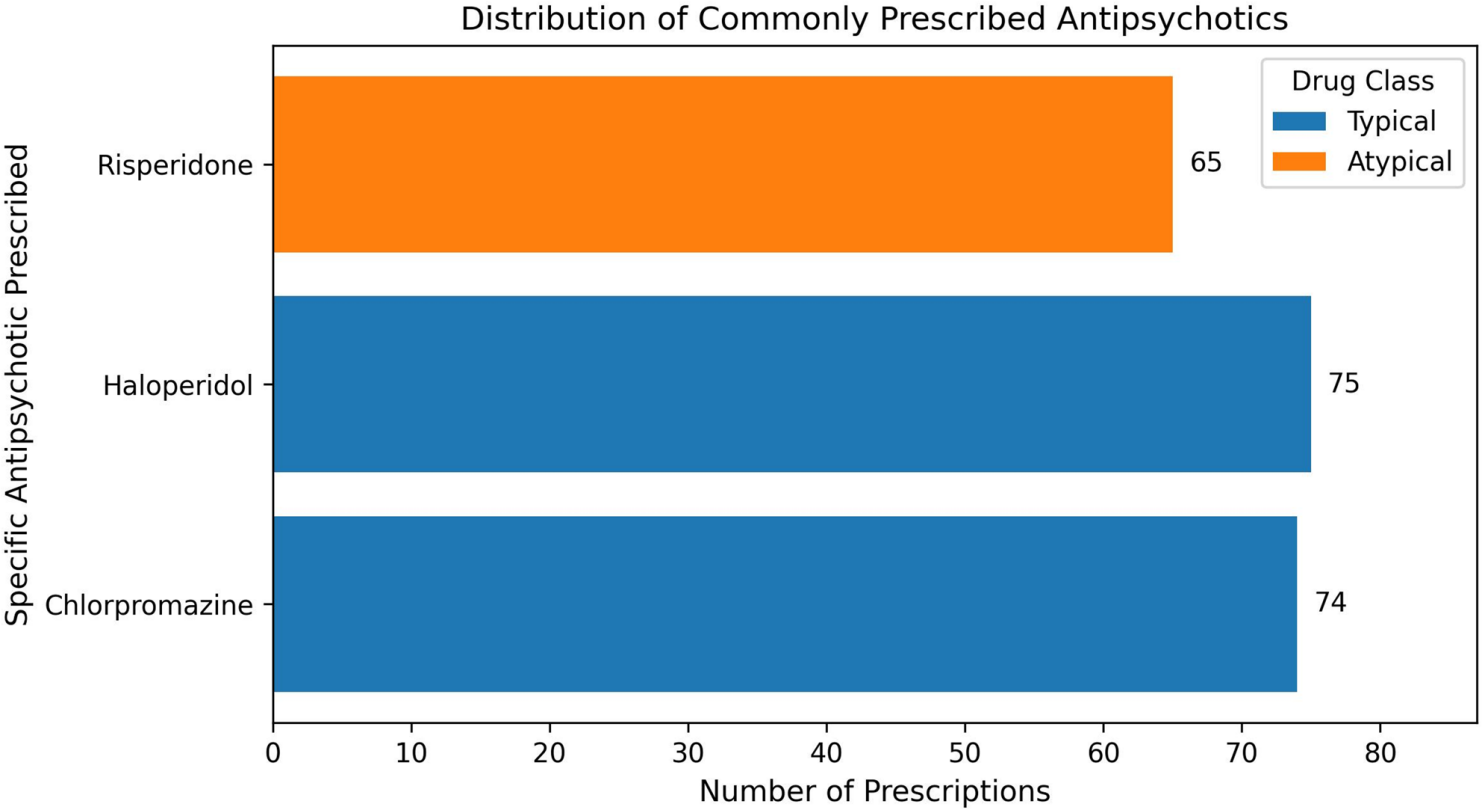
Distribution of Commonly Prescribed Antipsychotics

Figure 2 illustrates the distribution of commonly prescribed antipsychotics. Chlorpromazine (*n* = 74) and Haloperidol (*n* = 75), both first-generation (typical) agents, were the most frequently prescribed, followed by Risperidone (*n* = 65), a second-generation (atypical) agent. Color-coding highlights that typical antipsychotics accounted for a slightly larger share of prescriptions compared to atypical antipsychotics, indicating a relative preference for first-generation agents in the sample.

### Logistic regression analyses examining determinants of type of antipsychotic prescribing at a Zambian tertiary psychiatric unit

Table 2 summarizes the results of bivariate and multivariable logistic regression analyses examining determinants of antipsychotic prescribing. After adjustment for sociodemographic, clinical, and prescriber-related covariates, sex and medication adherence remained independently associated with the type of antipsychotic prescribed. Female patients had significantly higher odds of being prescribed a second-generation antipsychotic compared with male patients (AOR = 7.545; 95% CI: 3.270–17.410; *p* < 0.001). Partial adherence to medication was associated with significantly lower odds of SGA prescribing compared with complete adherence (AOR = 0.388; 95% CI: 0.164–0.920; *p* < 0.05).

**Table 2:** Bivariate and multivariable logistic regression results for determinants of antipsychotic prescribing

Predictor	Unadjusted				Adjusted			
	B	S.E.	Bivariate OR (95% CI)	*p*	B	S.E.	Multivariable AOR (95% CI)	*p*
**Age Group (Years)**								
18–24	Ref	Ref	Ref	Ref	Ref	Ref	Ref	Ref
25–34	0.702	0.398	2.018(0.925–4.404)	*P* > 0.05	0.127	0.487	1.135 (0.437–2.946)	*P* > 0.05
35–44	1.542	0.434	4.674(1.996–10.942)	< 0.001	0.333	0.601	1.395 (0.429–4.535)	*P* > 0.05
45+	1.516	0.490	4.554(1.742–11.906)	*P* < 0.05	0.465	0.680	1.593 (0.420–6.040)	*P* > 0.05
**Sex**								
Male	Ref	Ref	Ref	Ref	Ref	Ref	Ref	Ref
** Female**	**2.114**	**0.345**	**8.280 (4.214–16.269)**	**< 0.001**	**2.021**	**0.427**	**7.545 (3.270–17.410)**	**< 0.001**
**Marital Status**								
Single	Ref	Ref	Ref	Ref	Ref	Ref	Ref	Ref
Married	1.181	0.352	3.256 (1.633–6.492)	< 0.001	0.519	0.445	1.681 (0.702–4.022)	*P* > 0.05
Divorced/Widowed	1.464	0.468	4.325(1.728–10.821)	*P* < 0.05	0.417	0.637	1.517 (0.435–5.290)	*P* > 0.05
**Employment Status**								
Pupil/Student	Ref	Ref	Ref	Ref	Ref	Ref	Ref	Ref
Unemployed	1.657	0.759	5.242	*P* < 0.05	0.685	0.890	1.984 (0.347–11.350)	*P* > 0.05
Self-Employed/Employed	2.151	0.785	8.594	*P* < 0.05	1.161	0.944	3.193 (0.502–20.300)	*P* > 0.05
**Socioeconomic Status**								
Low (< ZMW 2,000)	Ref	Ref	Ref	Ref	Ref	Ref	Ref	Ref
Middle/High (≥ ZMW 2,000)	-0.391	0.350	0.466 (0.341-1.342)	< 0.001	-0.078	0.476	0.925 (0.364–2.350)	*P* > 0.05
**Primary Diagnosis**								
Brief/Acute & Transient Psychosis	Ref	Ref	Ref	Ref	Ref	Ref	Ref	Ref
Schizophrenia spectrum Psychosis	-0.544	0.530	0.580 (0.205–1.639)	*P* > 0.05	-0.245	0.663	0.782 (0.213–2.872)	*P* > 0.05
Other Types of Psychosis (Substance/Mood/Medical/PTSD/Other)	-0.487	0.322	0.614 (0.327–1.154)	*P* > 0.05	0.137	0.417	1.147 (0.506–2.599)	*P* > 0.05
**Prescribing Practitioner Specialty**								
Registrar – Psychiatry	Ref	Ref	Ref	Ref	Ref	Ref	Ref	Ref
Clinical Officer – Psychiatry (COP)/General (COG)	-0.941	0.272	1.194 (0.411–3.469)	< 0.001	-0.480	0.659	0.619 (0.170–2.252)	*P* > 0.05
**Prescriber Years of Experience in Mental Health**								
< 2 Years	Ref	Ref	Ref	Ref	Ref	Ref	Ref	Ref
2 to 5 Years	0.077	0.625	1.080 (0.317–3.679)	*P* > 0.05	0.053	0.725	1.055 (0.255–4.370)	*P* > 0.05
> 5 Years	0.017	0.639	1.017 (0.291–3.560)	*P* > 0.05	-0.276	0.767	0.759 (0.169–3.412)	*P* > 0.05
**Patient Adherence Level**								
Complete Adherence	Ref	Ref	Ref	Ref	Ref	Ref	Ref	Ref
Non-adherence	-1.050	0.658	0.350 (0.096–1.271)	*P* > 0.05	-0.617	0.724	0.540 (0.131–2.230)	*P* > 0.05
**Partial Adherence**	**-0.887**	**0.368**	**0.412 (0.200-0.848)**	*P* < 0.05	**-0.946**	**0.440**	**0.388 (0.164–0.920)**	*P* < 0.05

## Discussion

This study examined patterns and determinants of antipsychotic prescribing for first-episode psychosis (FEP) at Chainama Hills College Hospital in Lusaka, Zambia. We found that first-generation antipsychotics (FGAs)—particularly chlorpromazine and haloperidol—were prescribed more frequently than second-generation antipsychotics (SGAs), with risperidone being the most commonly prescribed SGA. Female sex was independently associated with higher odds of SGA prescribing, while partial adherence to treatment was associated with lower odds of receiving an SGA. Together, these findings provide important real-world evidence from a low-resource setting and underscore how FEP treatment is shaped by both clinical considerations and structural constraints.

Although nationally representative epidemiological data specific to first-episode psychosis in Zambia are limited, the sociodemographic profile observed in this study—characterized by young age at presentation, male predominance, high unemployment, and a high proportion of non-schizophrenia psychotic diagnoses—is consistent with reports from other Sub-Saharan African and low- and middle-income country early psychosis cohorts [3, 20, 21]. This suggests that, while derived from a single tertiary referral hospital, the sample reflects broader regional patterns of first-episode psychosis presentation.

The predominance of FGA prescribing observed in this study is consistent with prior reports from Sub-Saharan Africa and other low- and middle-income country (LMIC) settings [17, 20–22]. Although international guidelines generally recommend SGAs as first-line treatment for psychotic disorders, including early psychosis, these recommendations are based on nuanced trade-offs rather than unequivocal superiority [5, 9]. Meta-analyses and comparative effectiveness studies demonstrate that SGAs do not consistently outperform FGAs in symptom reduction, relapse prevention, or long-term functional outcomes, and that both classes are associated with high rates of early treatment discontinuation [7, 10]. Against this background, FGA predominance in this setting should not be interpreted simply as guideline discordance.

Rather, our findings reflect contextually rational prescribing shaped by health system realities. In Zambia, FGAs such as chlorpromazine and haloperidol are generally more available, more affordable, and more reliably included on national essential medicines lists than SGAs, making them the default option in resource-constrained settings [16, 17]. Prescriber familiarity also plays an important role, as FGAs have been used for decades and are well understood by clinicians working within constrained systems. In contrast, SGAs may be selectively prescribed when clinical judgment suggests a need for improved tolerability, when patients are perceived to be at higher risk of extrapyramidal symptoms, or when patients can afford the higher cost associated with SGAs [21, 22]. Similar patterns have been documented across the region, reinforcing that rational prescribing in LMICs may differ meaningfully from that in high-income contexts [20].

Sex differences in prescribing emerged as one of the most robust findings of this study. Female patients had significantly higher odds of receiving an SGA compared with male patients. This finding aligns with earlier research [23]. The pattern is consistent with extensive literature demonstrating that women are more susceptible to extrapyramidal symptoms (EPS) and tardive dyskinesia (TD) when treated with FGAs [6, 24]. Biological mechanisms—including estrogen-related modulation of dopaminergic pathways, greater dopamine receptor sensitivity, and sex-based differences in pharmacokinetics—contribute to higher effective drug exposure among women at equivalent doses, which increases their risk of developing movement disorders, akathisia, and hyperprolactinemia [6, 13, 25]. As a result, clinicians may preferentially select SGAs for female patients to mitigate movement disorder risk. A recent systematic review further highlight that sex differences emerge early in the course of psychosis and influence intervention onset, treatment response, and tolerability [26]. Although residual confounding by age or diagnostic category cannot be excluded, the observed association likely reflects clinically informed risk stratification rather than prescribing bias.

Partial adherence to antipsychotic treatment was independently associated with lower odds of SGA prescribing. While few studies have explicitly modeled adherence as a determinant of antipsychotic class, this pattern aligns with pragmatic clinical practice in low-resource settings. Non-adherence and partial adherence are common in psychosis and are strongly associated with relapse, rehospitalization, and clinical instability [18, 27]. Treatment guidelines and systematic reviews therefore recommend long-acting injectable antipsychotics (LAIs) as an adherence-enhancing strategy [10, 19]. However, in many LMIC settings—including Zambia—the most accessible LAIs are depot FGAs, whereas SGA LAIs are less consistently available and are often cost-prohibitive[17, 28]. Consequently, clinicians may preferentially manage partially adherent patients with FGAs as a pragmatic approach, while reserving SGAs for patients with more stable adherence or greater access to follow-up care. Importantly, non-adherence in psychosis is driven by a complex interplay of factors—including lack of illness insight, substance use, adverse attitudes toward medication, therapeutic alliance, and perceived benefits of treatment—rather than medication class alone [18]. High rates of non-adherence have also been documented among patients treated with SGAs [29], reinforcing that SGA prescribing alone does not resolve adherence challenges. From an implementation science perspective, these findings highlight how prescribing decisions reflect feasibility within existing systems rather than simple pharmacological preference. Taken together, our results emphasize the importance of interpreting antipsychotic prescribing patterns through a global mental health and implementation lens, recognizing that evidence-based recommendations must be adapted to local health system constraints.

### Implications for clinical practice, policy, and research

The findings of our study have clinical practice, policy, and research implications.

### Clinical practice

Clinicians managing FEP in resource-limited settings must balance evidence on efficacy and tolerability with medication availability, cost, patient adherence levels and alignment with established clinical guidelines [7, 8]. The observed sex differences in prescribing underscore the need for sex-sensitive prescribing practices and enhanced clinician awareness of differential adverse-effect risks [13, 24, 26]. Literature shows that women experience differential pharmacokinetics, slower metabolism of several psychotropic agents, and a higher propensity for adverse reactions such as hyperprolactinemia and weight gain [12, 13, 30–32]. This information should be included in the information education communication materials. Consequently, early integration of structured psychoeducation, family engagement, and adherence-support interventions may reduce relapse risk and improve long-term outcomes.

### Policy

At the policy level, these findings highlight the need to improve equitable access to antipsychotic medication options. Evidence from global essential-medicines studies shows that expanding affordable generic antipsychotic options on national essential medicines formularies, strengthening procurement and supply-chain systems significantly improve prescribing patterns and clinical outcomes [17, 33]. Policies that support pooled procurement, strategic purchasing, and inclusion of key SGAs on national essential-medicines lists can reduce stockouts and improve affordability [34–41]. The policies that align national treatment guidelines with implementation realities—rather than relying solely on high-income-country models—are critical for strengthening FEP care in LMICs.

### Future research

Future research should adopt prospective and longitudinal designs to capture key clinical and contextual factors not available in routine records, including symptom severity, duration of untreated psychosis, dosing, switching patterns, adverse effects, and clinical outcomes [3]. Qualitative studies examining prescriber decision-making and patient treatment experiences would provide deeper insight into contextual drivers of antipsychotic choice and guideline implementation in resource-limited settings [14]. Expanding investigations across multiple facilities, including district and community-based centers, would strengthen generalizability within Zambia and similar low-resource psychiatric contexts. Given the observed association between partial adherence and reduced SGA prescribing, future studies should evaluate adherence-focused interventions, such as structured psychoeducation, family engagement, and community follow-up, which have been shown to improve treatment continuity [42]. Research assessing the impact of system-level interventions, including procurement reforms and continuous professional development (CPD) programmes, on prescribing practices is also warranted [17, 43]. Finally, cost-effectiveness analyses comparing FGAs and SGAs would provide critical evidence to inform national treatment guidelines and procurement policies [16].

### Strengths and limitations

This study provides one of the few systematic examinations of antipsychotic prescribing patterns for first-episode psychosis (FEP) in Zambia, adding valuable evidence from a low-resource Sub-Saharan African setting. The use of routine clinical data collected over a five-year period allowed for inclusion of a diverse sample of patients treated in the country’s largest tertiary psychiatric referral hospital. The application of multivariable logistic regression enabled identification of independent determinants of antipsychotic prescribing while accounting for key sociodemographic, clinical, and prescriber-related factors.

Several limitations should be considered when interpreting these findings. First, the retrospective cross-sectional design precludes causal inference and limits assessment of temporal relationships between patient characteristics and prescribing decisions. Second, data quality depended on the completeness and accuracy of medical records. Although a standardized data abstraction form was used (Supplementary Table A), several clinically important variables—including symptom severity at presentation, duration of untreated psychosis, dosing, polypharmacy, medical and substance use comorbidities, adverse effects, and hospitalizations related to treatment—were inconsistently documented or unavailable and could not be included in the analyses. As a result, unmeasured confounding cannot be ruled out. This calls for the need for standardized measures that can capture information regarding FEP in a more comprehensive manner so that patients are managed in a holistic manner both at initiation of medication and follow up of treatment. Third, antipsychotic prescribing and adherence were assessed during the initial treatment period following first antipsychotic initiation. Medication adherence was based on clinician-documented evaluations derived from inpatient observation and/or early outpatient follow-up and therefore reflects early treatment adherence rather than longer-term adherence trajectories. Only antipsychotic prescriptions initiated following documentation of first-episode psychosis, and the corresponding primary DSM-5/ICD-10 diagnosis were analyzed, and only the initial prescription was included. Consequently, changes in treatment over time, including switching and discontinuation, were not captured. Fourth, the study was conducted at a single tertiary referral hospital, which may limit generalizability to district-level or community-based mental health settings in Zambia. Fifth, the “Other Types of Psychosis” diagnostic category encompasses heterogeneous conditions, including both organic and nonorganic psychotic disorders. Subclass analyses were not conducted due to limited sample sizes for individual diagnoses, which would have reduced statistical power and interpretability. Future studies with larger samples should examine whether outcomes differ across specific psychosis subtypes. Finally, potentially relevant factors such as patient preferences, prescriber attitudes, and broader regulatory or formulary constraints were not systematically captured in routine records, restricting the scope of analysis. These limitations highlight the need for improved standardization of clinical documentation and for prospective, multi-site studies to strengthen the evidence base.

## Conclusion

This study provides one of the few examinations of antipsychotic prescribing patterns and their determinants for first-episode psychosis in a tertiary psychiatric hospital in Zambia. First-generation antipsychotics remain the predominant treatment option, with prescribing decisions influenced by patient sex and treatment adherence. Importantly, these findings indicate that prescribing practices in low-resource settings reflect pragmatic, context-driven decision-making shaped by medication availability, affordability, and health system constraints, rather than simple departures from clinical guidelines. Strengthening first-episode psychosis care will require coordinated efforts to improve access to effective antipsychotic options, integrate sex-sensitive and adherence-focused interventions, and promote continuing professional development for prescribers. By situating early pharmacological decisions within their clinical and structural context, this study contributes contextually grounded evidence to the global mental health literature and provides a foundation for future system-level improvements in first-episode psychosis care.

## Supplementary Information

Below is the link to the electronic supplementary material.

Supplementary Material 1

## Data Availability

The datasets generated and/or analyzed during the current study are not publicly available due to ethical and confidentiality restrictions. Data may be made available from the corresponding author on reasonable request and with prior approval from an accredited ethics review committee and other relevant authorities in Zambia.

## Abbreviations

FEP: First Episode Psychosis
FGAs: First Generation Antipsychotics
SGAs: Second Generation Antipsychotics
CPD: Continuous Professional Development
LAIs: Long-acting Injectable Antipsychotics
TD: Tardive Dyskinesia
EPS: Extrapyramidal Symptoms
