# Supplementary material for: Antipsychotic prescribing patterns and determinants in first-episode psychosis: a 2019–2024 cross-sectional study from Zambia
Source: BMC Psychiatry. 2026 Jan 30;26:208. doi: 10.1186/s12888-026-07847-y (PMC12931020; doi:10.1186/s12888-026-07847-y)
Supplement: Supplementary file 1 — Supplementary Material 1 [file 12888_2026_7847_MOESM1_ESM.docx]

**Supplementary Table A**

**Data Extraction Form utilized for the study**

**Section A: Patient Demographics**

| **Description** | **Finding** |
| --- | --- |
| 1. Patient ID/Record Number/ Study Number |  |
| 1. Age at First Episode: |  |
| 1. Gender: | ☐ Male  ☐ Female |
| 1. Marital Status: | ☐ Single  ☐ Married  ☐ Divorced  ☐ Widowed |
| 1. Employment Status: | ☐ Formally Employed  ☐ Self Employed  ☐ Unemployed  ☐ Student  ☐ Retired |
| 1. Socioeconomic Status: | ☐ Low (< ZMW 2,000)  ☐ Middle (ZMW 2,000–9,200)  ☐ High (> ZMW 9,200) |

**Section B: Clinical Information**

| **Description** | **Finding** |
| --- | --- |
| 1. Date of First Psychotic Episode: |  |
| 1. Primary Diagnosis (DSM-5/ICD-10): | ☐ Schizophrenia  ☐ Schizoaffective Disorder  ☐ Bipolar Disorder with Psychotic Features  ☐ Brief Psychotic Disorder  ☐ Major Depressive Disorder with ☐ Psychosis  ☐ Other (specify): ___________________ |
| 1. Severity of Symptoms at Presentation: | ☐ Mild  ☐ Moderate  ☐ Severe |
| 1. Comorbidities: | ☐ Substance Use Disorder  ☐ Primary Psychiatric Disorder (Depression etc)  ☐ Another Medical Condition (HIV/AIDs etc) |

**Section C: Treatment Information**

| **Description** | **Finding** |
| --- | --- |
| 1. Type of Antipsychotic Prescribed: | ☐ First-generation (typical)  ☐ Second-generation (atypical)  ☐ Both First- and Second-Generation antipsychotics |
| 1. Specific Antipsychotic(s) Prescribed: |  |
| 1. Dosage of Antipsychotic(s): |  |
| 1. Duration of Treatment (in weeks): |  |
| 1. Patient Adherence to Treatment: | ☐ Complete Adherence  ☐ Partial Adherence  ☐ Non-Adherent |

**Section D: Practitioner Information**

| **Description** | **Finding** |
| --- | --- |
| Prescribing Practitioner’s Specialty: | ☐ Psychiatrist  ☐ Registrar  ☐ Senior resident Medical Officer  ☐ Clinical Officer (General)  ☐ Clinical Officer (Psychiatry)  ☐ Medical Licentiate |
| **Years of Experience in mental health of Prescribing Practitioner:** | ☐ Less than 2 years  ☐ 2-5 years  ☐ Greater than 5 years |

**Section E: Outcome Information After Treatment with Prescribed Medication**

| **Description** | **Finding** |
| --- | --- |
| 1. Clinical Outcome After Treatment (at follow-up): | ☐ Full Remission  ☐ Partial Response  ☐ Stable but symptomatic  ☐ Minimal Improvement  No Improvement  Worsening  Side-effects  Discontinued |
| 1. Adverse Effects Reported: | Extrapyramidal Symptoms (EPS):  Metabolic Side Effects  Sedation and Cognitive Effects  Cardiovascular Effects  Endocrine Effects:  Anticholinergic Effects:  Haematological Effects:  Neurological Effects:  Other Effects: |
| 1. Hospitalizations Due to Treatment (if any): | ☐ Yes  ☐ No  If Yes, specify duration and reason: ________ |
